# Plasma Neurofilament Light Is Not Associated with Ongoing Neuroaxonal Injury or Cognitive Decline in Perinatally HIV Infected Adolescents: A Brief Report

**DOI:** 10.3390/v14040671

**Published:** 2022-03-24

**Authors:** Julie van der Post, Jason G. van Genderen, Johannes A. Heijst, Charlotte Blokhuis, Charlotte E. Teunissen, Dasja Pajkrt

**Affiliations:** 1 Pediatric Infectious Diseases, Emma Children’s Hospital, Amsterdam UMC Location University of Amsterdam, 1105 Amsterdam, The Netherlands; j.g.vangenderen@amsterdamumc.nl (J.G.v.G.); c.blokhuis@amsterdamumc.nl (C.B.); d.pajkrt@amsterdamumc.nl (D.P.); 2 Neurochemistry Laboratory and Biobank, Department of Clinical Chemistry, Amsterdam UMC Location Vrije Universiteit Amsterdam, 1117 Amsterdam, The Netherlands; h.heijst@amsterdamumc.nl (J.A.H.); c.teunissen@amsterdamumc.nl (C.E.T.)

**Keywords:** NfL, HIV, pathogenesis, brain structure, cognitive performance, neuroaxonal injury

## Abstract

Despite combination antiretroviral therapy (cART), adolescents with perinatally acquired human immunodeficiency virus (PHIV) exhibit cerebral injury and cognitive impairment. Plasma neurofilament light (pNfL) is a biomarker identified as a promising marker associated with neuroaxonal injury and cognitive impairment. To investigate whether cerebral injury in cART-treated PHIV adolescents is persistent, we longitudinally measured pNfL. We included 21 PHIV adolescents and 23 controls, matched for age, sex, ethnic origin and socio-economic status. We measured pNfL in both groups and CSF NfL in PHIV adolescents using a highly sensitive Single Molecule Array (Simoa) immunoassay. We compared pNfL between groups over time with a mean follow-up time of 4.6 years and assessed its association with MRI outcomes, cognitive function and HIV-related characteristics using linear mixed models. The median age was 17.5 years (15.5–20.7) and 16.4 years (15.8–19.6) at the second assessment for PHIV adolescents and controls, respectively. We found comparable pNfL (PHIV vs. controls) at the first (2.9 pg/mL (IQR 2.0–3.8) and 3.0 pg/mL (IQR 2.3–3.5), *p* = 0.499) and second assessment (3.3 pg/mL (IQR 2.5–4.1) and 3.0 pg/mL (IQR 2.5–3.7), *p* = 0.658) and observed no longitudinal change (coefficient; −0.19, 95% −0.5 to 0.1, *p* = 0.244). No significant associations were found between pNfL and HIV- or cART-related variables, MRI outcomes or cognitive function. We observed low CSF NfL concentrations at the baseline in PHIV adolescents (100.8 pg/mL, SD = 47.5). Our results suggest that there is no ongoing neuroaxonal injury in cART-treated PHIV adolescents and that the neuroaxonal injury is acquired in the past, emphasizing the importance of early cART to mitigate HIV-related neuroaxonal damage.

## 1. Introduction

Combination antiretroviral therapy (cART) improved the survival rates of effectively treated patients living with perinatally acquired HIV (PHIV) [1,2]. Subsequently, the incidence of severe HIV-associated neurological complications, e.g., HIV-encephalopathy, decreased [3].

Despite adequate cART, available literature suggests persistent lower white matter (WM) integrity, lower intelligence quotient (IQ) and impaired executive function in PHIV children and adolescents compared to healthy controls [4,5,6]. The pathogenesis of neurological and cognitive complications in PHIV children and adolescents is not fully understood. Proposed hypotheses include HIV-induced neuroinflammation, early neurological damage prior to treatment initiation, ongoing viral replication in the central nervous system (CNS), neurotoxic effects of cART and confounding effects such as socio-economic factors [7,8,9].

Several biomarkers of inflammation, immune activation, coagulation and neuronal degeneration have been previously studied and sometimes found to be elevated or associated with neurologic outcomes in (cART-treated) PHIV children and adolescents [8,9,10,11,12,13,14,15,16]. Neurofilament light (NfL)—a protein released upon neuroaxonal injury into the cerebrospinal fluid (CSF) and plasma—has been identified as a promising biomarker for diagnostic and prognostic purposes, as it corresponds with neuroaxonal injury and degeneration in other neurodegenerative diseases such as pediatric multiple sclerosis, acute disseminated encephalomyelitis, spinal muscular atrophy and perinatal asphyxia [17,18,19].

In adults with HIV, CSF NfL is elevated compared to controls and NfL in CSF or plasma is sometimes associated with cognitive impairment, however, associations and differences are often driven by untreated participants, lower CD4^+^ T-cell lymphocyte counts or higher HIV viral load [20,21,22,23,24]. cART has a positive effect on HIV-related neuroaxonal injury, as lower CSF NfL or pNfL concentrations were found in cART-treated HIV-infected adults compared to adults without adequate viral suppression [20,21,22,23]. Studies on NfL in PHIV children are scarce and almost solely cross-sectional and, therefore, do not evaluate disease progression over time [13,14,15]. The majority of studies either found similar serum or plasma NfL (pNfL) concentrations in PHIV children compared to the controls or did not report a significant change in concentration over time in PHIV children [13,14,15,16]. To further understand the pathophysiologic mechanisms of HIV-associated neurological and cognitive impairment in PHIV children and adolescents despite cART treatment, we assessed NfL as a pathological substrate of ongoing cognitive impairment and neuroaxonal damage in our neurological, cognitive and visual performance in the perinatally HIV-infected children (NOVICE) cohort of cART-treated PHIV adolescents with persistent lower WM integrity and decreased executive function, previously observed in longitudinal studies [25,26]. We, therefore, compared pNfL concentrations between cART-treated PHIV adolescents and matched controls at the baseline and follow-up, and explored associations with structural cerebral markers and cognitive function in the PHIV group over time.

## 2. Materials and Methods

This brief report is partly a NOVICE study: a cohort study investigating neurological, cognitive, ophthalmological and cardiovascular impairment in cART-treated PHIV adolescents compared to healthy controls matched for age, sex, ethnic origin and socio-economic status (SES).

### 2.1. Study Participants

Between December 2012 and January 2014, PHIV adolescents visiting the outpatient clinic of the Emma Children’s Hospital—part of the Academic Medical Center (AMC)—were invited to participate in the NOVICE study. Healthy controls were recruited through schools, sports clubs and churches in Amsterdam [27]. Between February 2017 and July 2018, all participants were approached to participate in the second assessment, and we used exclusion criteria as previously described [25]. To ensure equal distribution and reduce the risk of known confounding variables, groups were also matched for body mass index (BMI) [28,29,30]. Informed consent was obtained from all parents or legal guardians and participants aged 12 years and above. We adhered to the tenets of the Declaration of Helsinki and obtained approval from the Ethics Committee of the AMC. The study is registered at the Dutch Trial Registry (identifier: NL6813).

### 2.2. Plasma NfL and CSF NfL

Blood samples were collected through venipuncture at first and second assessment and a subset of PHIV children underwent a lumbar puncture at first assessment to obtain CSF as previously reported [13]. Plasma and CSF samples were stored at −80 °C until time of measurement. CSF and pNfL concentrations were measured with a Single Molecule Array (Simoa) NF-light^TM ®^ Advantage Kit run on automated HD-X Analyzer (Quanterix, Lexington, MA, USA) [31]. Upper CSF NfL reference values were calculated with the following formula: CSF NFL = 210.22 × 1.031^age^ [32].

### 2.3. Demographics, Disease and Treatment-Related Characteristics

Historical HIV and treatment-related characteristics were identified through patient record data and provided by Sichting HIV Monitoring (SHM, HIV Monitoring Foundation, Amsterdam, The Netherlands). Demographic data were collected through questionnaires. We repeated an HIV test during the second assessment to re-confirm their HIV-negative status in controls. In this study, cART was defined as the use of at least three antiretroviral drugs from a minimum of two drug classes.

### 2.4. Structural Cerebral Variables for Association Analysis

We previously reported magnetic resonance imaging (MRI) acquisition and processing details [26]. In summary, a 3-Tesla MRI (Intera, Philips Healthcare, Best, The Netherlands) was repeated in all participants. T1-weighted images with magnetization-prepared rapid acquisition gradient echo (MPRAGE) was used to determine gray matter (GM) and WM volumetric analysis. Fluid-attenuated inversion recovery (FLAIR) sequence was used to determine macroscopic WM lesions, i.e., WM hyperintensities (WMH) volume. Diffusion tensor imaging (DTI) was used to analyze WM integrity. We used the following variables WM integrity markers: fractional anisotropy (FA), mean diffusivity (MD), axial diffusivity (AD) and radial diffusivity (RD), as previously described [26].

### 2.5. Cognitive Function Variables for Association Analysis

A neuropsychological assessment was performed by an experienced neuropsychologist who was unaware of participants’ HIV status. A test battery—identical in both assessments—was used to determine intelligence quotient (IQ) and various cognitive domains, including processing speed, learning ability, visual–motor function, working memory and executive function [25]. Variables of cognitive function that were previously defined and investigated were used for association analysis [25].

### 2.6. Statistical Analysis

We conducted statistical analysis in R version 4.0.3 and R Studio version 1.4.1 R Core Team (2020, R Foundation for Statistical Computing, Vienna, Austria) [33]. We explored selective dropout by comparing pNfL, demographic characteristics and neurocognitive outcome on first assessment, between participants and dropouts for longitudinal analysis [34]. We compared demographic, HIV and cART-related characteristics using the unpaired *t*-test or Mann–Whitney *U* test for normally or non-normally distributed continuous data, respectively, and the Fisher’s exact test for categorical data. If necessary for statistical analysis, non-normally distributed variables were logarithmically transformed prior to analysis. We used linear mixed models (package: lmer) to assess changes in pNfL over time with the group by time interactions and associations between pNfL and neuro-imaging outcomes and cognitive domain scores. We explored associations between pNfL and HIV- and treatment-related variables, i.e., duration of cART use, CDC stage, nadir CD4^+^ T-cell *Z* score, zenith HIV viral load (VL), age at diagnosis and age at treatment initiation. We adjusted analyses for age, sex and BMI [28,29,30]. In addition, we used linear mixed models (package: lmer) to explore associations between integrase strand transfer inhibitors (INSTIs), protease inhibitors (PIs) and non-nucleoside reverse transcriptase inhibitors (NNRTIs) and pNfL concentrations and variables of cognitive function. We adjusted analyses for age and sex for variables of cognitive function and additionally for BMI for pNfL association analysis [28,29,30]. A *p*-value < 0.05 was considered statistically significant. Adjustment for multiple comparisons such as Bonferroni correction was not performed, as this study was designed as exploratory [35]. Finally, we performed a sensitivity analysis with and without any outliers affecting both results and assumptions, to determine the influence on primary and secondary outcomes.

## 3. Results

### 3.1. Characteristics of Study Participants

In total, 21/36 (58%) PHIV participants and 23/37 (62%) matched controls from the first assessment provided consent for the longitudinal assessment. Reasons for not participating included a disinclination to participate (nine in both groups), an inability to reach (five controls) or a relocation (two PHIV). Two PHIV children were excluded from association analysis due to insufficient sample volumes. There was no selective dropout. Median age (in years) at the second assessment was 17.5 (IQR 15.5–20.7) in PHIV adolescents and 16.4 (IQR 15.8–19.5) for controls. More PHIV children were born in sub-Saharan Africa compared to controls (*p* < 0.001). We found no other differences in the characteristics of study participants (Table 1). Different cART-treatment regimens are presented in Supplementary Materials Table S2.

### 3.2. Plasma NfL Concentrations over Time and CSF NfL at Baseline

**Figure 1:**
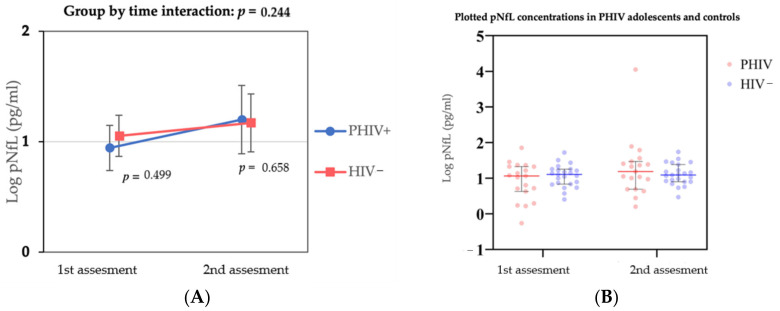
pNfL concentrations (pg/mL) at different timepoints between PHIV and HIV- participants and group by time interaction. (**A**) Group by time interaction of log-transformed pNfL concentrations between PHIV participants and HIV− controls. Values are plotted of least square means and adjusted for age, sex and BMI. (**B**) Individual plotted log-transformed pNfL concentrations (pg/mL) at different timepoints between PHIV participants and HIV− controls, with median time between assessment 4.6 years (IQR 4.4–4.8).

We found a positive association between age and sNfL in the cross-sectional analysis (coefficient; 0.04, 95% CI 0.008—0.07, *p* = 0.017), adjusted for sex and BMI. We found no significant difference in pNfL concentration changes over time in both groups (coefficient; −0.19, 95% −0.50 to 0.12, *p* = 0.244) nor between groups at the first (*p* = 0.499) and second assessment (*p* = 0.658) (Figure 1). At the first assessment, median pNfL concentration was 2.9 pg/mL (IQR 2.0–3.8) and 3.0 pg/mL (IQR 2.3–3.5), respectively, for PHIV participants and controls. At the second assessment, median pNfL was 3.3 pg/mL (IQR 2.5–4.1) for PHIV participants and 3.0 pg/mL (IQR 2.5–3.7) for controls (Table 2). A sensitivity analysis excluding one outlier (pNfL 57.4 pg/mL) did not change the findings (data not shown). Mean CSF NfL concentration at baseline in PHIV children was 100.84 pg/mL (SD = 47.5), which was 33-fold higher than pNfL. We found no significant association between NfL concentrations in CSF and plasma (R^2^; 0.16, β-coefficient; 0.291, 95% −0.213–0.795, *p*-value = 0.240), and CSF NfL concentrations were below the aforementioned suggested upper reference values (CSF NFL = 210.2 × 1.031^age^) (data not shown).

### 3.3. Associations between pNfL and Neurocognitive Outcomes and Disease, Treatment-Related Variables and CSF NFL

We found no significant associations between pNfL and any of the neuroimaging markers or cognitive function domains. In the PHIV group, pNfL was not associated with any parameters relating to HIV disease severity or treatment (Supplementary Materials Table S1). No significant associations were observed between different cART regimens and pNfL or variables of cognitive function (Supplementary Materials Tables S2 and S3).

## 4. Discussion

In this study, we assessed the role of pNfL, a marker for neuroaxonal damage, as a substrate for ongoing neuroaxonal damage and cognitive impairment to further understand the pathophysiologic mechanisms of HIV-associated neurologic and cognitive impairment in PHIV children despite effective treatment. Baseline plasma NfL (in both groups) and CSF NfL concentrations (in the PHIV group) were within the suggested reference range. Over time, both groups showed similar changes in pNfL concentrations. Furthermore, no associations were observed between pNfL and imaging outcomes, cognitive function and disease- or treatment-related variables.

The low NfL levels may, in part, be explained by treatment. Our results are in line with previous cross-sectional studies in PHIV children and adolescents on adequate cART treatment [13,14,15]. Furthermore, in HIV-infected adults with higher CSF NfL or pNfL concentrations compared to healthy controls, NfL decreased or even normalized after cART initiation [20,21,22,23]. Correspondingly, lower pNfL concentrations were observed in PHIV children on cART [15]. This supports the hypothesis that HIV-associated neuroaxonal injury may be partially mitigated by effective cART, and emphasizes the importance of early and effective treatment. Moreover, these results might suggest cART-related neurotoxicity is an unlikely contributor to neuroaxonal injury. This is further substantiated by our assessment on different cART regimens, in which no associations were observed between cART regimens and pNfL. However, this should be interpreted with caution, as CNS penetration and neurotoxic effects of different cART regimens remain incompletely understood, and evidence suggests cART-related neuroinflammation and neurologic and psychiatric side effects of specific antiretroviral agents, such as Efavirenz [36].

The lack of associations between NfL and neuroimaging and neurocognitive outcomes contrasts with findings in several other studies in cART-treated adults [20,22,23]. This difference between findings in adult and pediatric populations could point towards a possible difference in the pathogenesis of neurocognitive impairment in perinatal versus adult infection. In children, early neuroinflammation and neuronal damage might interfere with CNS development [1,9]. This may be a more significant mechanism in the pathogenesis of neurocognitive outcomes in PHIV children than ongoing neuroinflammation. In our cohort, we previously reported decreased executive functioning in PHIV adolescents, but otherwise similar cognitive function and structural brain development over time compared to matched controls [25,26]. As pNfL concentrations remained stable during the follow-up period, we hypothesized the decreased executive function to be a persistent effect of HIV-related CNS damage acquired prior to treatment initiation, rather than ongoing neuroaxonal injury. Otherwise, the lack of severe decline in cognitive function or neuroimaging abnormalities over time in our cohort may have further hampered the detection of possible associations with pNfL- and PHIV-associated neuronal injury or cognitive impairment.

Alternatively, the minor NfL variations within the normal range may lack sensitivity to detect neuroaxonal injury in our cohort of treated PHIV children. Earlier cross-sectional analysis of CSF biomarkers in the PHIV children in our cohort suggested the presence of low-grade neuroinflammation despite adequate viral suppression [13]; however, this was not reflected by NfL levels over time in the current study. NfL may better represent neuroaxonal injury in more severe stages of neurological disease. This is demonstrated in the literature in HIV adults, where elevated CSF NfL is associated with HIV-associated dementia, severe forms of HIV-associated cognitive disease (HAND) or driven by HIV participants without cART when associated with milder forms of HAND [20,21,23]. This is supported by the finding that associations with cognitive impairment and structural brain injury appeared driven by untreated PHIV children and adolescents [15]. In most adult studies, significant associations between higher pNfL or CSF NfL and HIV-associated neurocognitive disease are also primarily driven by or exclusively found in participants that are untreated or have lower CD4^+^ T-cell lymphocyte counts and a higher HIV viral load [20,22,23,24].

Another explanation for the discrepancy in the findings between adult and pediatric populations may be confounding. Many studies in adults use univariate analyses or do not adjust for sex, age or BMI. In addition, associations found in other studies with pNfL or CSF NfL could otherwise be explained by additional factors and variation in study demographics potentially influencing cognitive decline, brain development or higher pNfL values, such as physical sports with a risk of head trauma and even sleep deprivation [1,7,29].

Lastly, no significant association was observed between CSF NfL and plasma NfL, in contrast with previously published data on neurodegenerative diseases and adult HIV [19,29,32]. A possible explanation is the low NfL concentration in both plasma and the CSF of PHIV participants at baseline. In a previous study in patients with amyotrophic lateral sclerosis, high concentrations of CSF NfL and pNfL are significantly correlated, while there was no correlation between low plasma and CSF NfL concentrations in the healthy control group [37].

This brief report uniquely reports on longitudinal pNfL assessment in PHIV adolescents and well-matched controls for age, sex, ethnic background and socioeconomic status, with long-term follow-up. We additionally controlled for known confounders such as BMI, as lower BMI is associated with higher pNfL concentrations [28,29]. This brief report has some limitations. Overall, the small cohort size may have led to type II errors and reduced the generalizability [38]. NfL is not disease-specific and is related to neuroaxonal injury in multiple neurodegenerative diseases of both the central and peripheral nervous systems. Finally, this study was designed as exploratory and results should, therefore, be interpreted with caution, since no correction was applied for multiple testing.

## 5. Conclusions

In conclusion, when evaluated over time, PHIV children and adolescents on cART and matched uninfected controls have comparable low pNfL concentrations. No associations were found between pNfL and parameters of structural brain injury or cognitive function. Our findings suggest the absence of ongoing NfL-sensitive neuroaxonal injury in PHIV adolescents.

## Figures and Tables

**Table 1:** Study participants’ characteristics.

Demographics	PHIV (*n* = 21)	Controls (*n* = 23)	*p*-Value
Age (y) at first assessment	13.4 (10.9–15.6)	12.1 (11.1–15.2)	0.655 ^X^
Age (y) at second assessment	17.5 (15.5–20.7)	16.38 (15.8–19.6)	0.526 ^X^
Sex (Female)	9 (43%)	14 (61%)	0.365 ^Z^
Follow-up time (y)	4.60 (4.4–4.8)	4.60 (4.4–4.8)	0.284 ^X^
Country/Region of birth, no. (%)			
The Netherlands Sub-Sahara Africa Other	5 (24%) 13 (62%) 3 (14%)	22 (96%) 1 (4%) 0 (0%)	<0.001 ^Z^*
Ethnic origin, no (%)			
Black White Other	17 (81%) 0 (0%) 4 (19%)	18 (78%) 2 (9%) 3 (13%)	0.744 ^Z^
BMI (kg/m^2^)			
First assessment Second assessment	18.4 (17.3–20.3) 20.4 (19.2–22.3)	19.7 (17.7–21.8) 22.2 (19.9–26.3)	0.051 ^#^ 0.065 ^#^
Systolic blood pressure (mmHg)			
First assessment Second assessment	110 (100–112) 120 (114–132)	105 (95–110) 120 (113-124)	0.264 ^#^ 0.569 ^#^
Diastolic blood pressure (mmHg)			
First assessment Second assessment	65 (55–72) 67 (59–76)	65 (65–71) 64 (60–73)	0.041 ^#^ 0.823 ^#^
Lifestyle, no. (%)			
Ever smoked, first assessment Ever smoked, second assessment	1 (5%) 7 (33%)	0 (0%) 5 (22%)	0.452 ^Z^ 0.363 ^Z^
**HIV and cART-related variables**			
Age at HIV diagnosis (y)	1.72 (0.83–4.16)	-	-
CDC Stage:			
N or A B C	8 (40%) 8 (35%) 5 (25%)	-	-
Zenith HIV VL (^10^log copies/mL)	5.54 (5.0–5.8)	-	-
Nadir CD4+ T-cell *Z* score	−0.70 (−1.2 to −0.39)	-	-
Age at cART initiation (y)	2.5 (1.2-5.97)	-	-
HIV diagnosis to cART initiation (y)	0.3 (1.3–0.8)	-	-
Duration cART use (y)	14.9 (9.5–19.6)	-	-
cART use at second assessment	19 (95%)		
Undetectable HIV VL at second assessment	17 (90%)	-	-
Undetectable HIV VL between first and second assessment	13 (70%)		

Abbreviations: Values are reported as median (IQR = interquartile range, third–first quartile) or no. (%). CD4+ T-cell presented in Z-scores adjusted for mean appropriate age and HIV- controls; cART = combination antiretroviral therapy; CDC = Centers for Disease Control and Prevention, classification system used to classify HIV- stage (N = no symptoms, A = minimal symptoms, B = moderate symptoms, C = severely symptomatic/immunodeficiency syndrome); kg = kilogram; m = meter; *n* = number, PHIV = perinatally acquired human immunodeficiency virus; undetectable viral load is defined as ≤150 copies/mL including viral blips; VL = viral load; y = year; Zenith VL values extracted for duration of follow-up and transformed using 10-base logarithm prior to analysis; statistical tests: X = Mann–Whitney *U* test; Z = Fisher’s exact test; # adjusted *p*-values for age and sex; * *p* < 0.05. Deviation in number (PHIV): Zenith HIV VL (*n* = 18), Nadir CD4+ T-cell (*n* = 19), Treatment initiation and duration (*n* = 18).

**Table 2:** Neurofilament light (NfL) concentrations in plasma and cerebrospinal fluid and group by time interaction effect.

	PHIV (*n* = 21)	HIV− (*n* = 23)	Coefficient (95% CI)	*p*-Value
pNfL (pg/mL)				
at first assessment at second assessment	2.9 (2.0–3.8) 3.3 (2.5–4.1)	3.0 (2.3–3.1) 3.0 (2.5–3.7)	-	0.499 ^X^ 0.658 ^X^
CSF NfL (pg/mL)				
at first assessment	100.84 (47.5)	-	-	-
Group × Time interaction log pNfL ^Z^	-		−0.19 (−0.50–0.12)	0.244 ^Y^

Abbreviations: pNfL, plasma Neurofilament light; CSF, cerebrospinal fluid; Z, group by time interaction; difference in change of pNfL concentrations over time in both groups with pNfL concentrations transformed using natural logarithm prior to analysis, 95% CI = 95% confidence interval; *n* = number; statistical tests: X = Mann–Whitney *U* test; Y = linear mixed models with *p*-value adjust for age, sex and BMI.

## Data Availability

Data cannot be shared publicly because the data contain (potentially) sensitive patient information. Data is available (in anonymous form) upon request to the corresponding author.

## Supplementary Materials

The following supporting information can be downloaded at: https://www.mdpi.com/article/10.3390/v14040671/s1, Table S1, Associations between pNfL and neuroimaging, cognitive and disease and treatment related variables, Table S2, Different cART regimens at first and second assessment in PHIV adolescents, Table S3, Different cART regimens and association with pNfL and cognitive function variables over time.
