# Supplementary material for: Plasma Neurofilament Light Is Not Associated with Ongoing Neuroaxonal Injury or Cognitive Decline in Perinatally HIV Infected Adolescents: A Brief Report
Source: Viruses. 2022 Mar 24;14(4):671. doi: 10.3390/v14040671 (PMC9030750; doi:10.3390/v14040671)
Supplement: Supplementary file 1 [file viruses-14-00671-s001.zip › viruses-1620918-supplementary.pdf]

Supplementary Table S1. Associations between pNfL and neuroimaging, cognitive and disease and treatment related variables

	plasma Neurofilament Light (pNfL)	
Variables association analysis	Coefficient (95% CI)	Adjusted *p*-value
**Neuroimaging**		
White matter volume #	0.01 (-0.006 – 0.018)	0.351
Grey matter volume	0.001 (-0.008 – 0.011)	0.773
White matter hyperintensities #	-0.17 (-1.63 – 1.33)	0.828
Mean diffusivity #	-0.002 (-0.012 – 0.007)	0.750
Axial diffusivity #	-0.003 (-0.014 – 0.005)	0.455
Fractional anisotropy #	-0.003 (-0.004 – 0.010)	0.395
Radial diffusivity #	-2.40 (-7.01 – 2.13)	0.323
**Cognitive function**		
Executive function #	0.33 (-0.43 – 1.11)	0.412
Processing speed #	-1.06 (-6.29 – 4.38)	0.698
Learning ability #	0.296 (-0.14 – 0.75)	0.200
Visual motor function	0.09 (-0.01 – 0.20)	0.091
Working memory #	-0.32 (-1.36 – 0.73)	0.548
Intelligence quotient #	1.7 (-0.02 – 0.06)	0.143
**HIV- and cART related variables**		
CDC-C	-0.06 (-0.24 – 0.12)	0.536
Age at diagnosis	0.02 (-0.07 – 0.11)	0.676
Zenith HIV VL #	0.29 (-0.353 – 0.938)	0.599
Nadir CD4+ T-cell	0.06 (-0.33 – 0.46)	0.288
Duration of cART (y)	0.01 (-0.07 – 0.10)	0.823
Age at start cART (y)	-0.04 (-0.08 – 0.07)	0.960
Undetectable HIV VL at second assessment	1.33 (-7.18 – 9.83)	0.780
Undetectable HIV VL between first and second assessment	1.9 (-5.45 – 9.31)	0.631

Linear mixed model formulas were used for longitudinal association analysis adjusted for age, sex and BMI; # These variables were transformed using natural logarithm prior to analysis. AIDS = acquired immunodeficiency syndrome; cART = combination antiretroviral therapy; CI = 95% confidence interval; CDC = Centers for Disease Control and Prevention, classification system used to classify HIV- stage (N = no symptoms, A = minimal symptoms, B = moderate symptoms, C = severely symptomatic/immunodeficiency syndrome); VL = viral load; y = year; Zenith VL values extracted for duration of follow-up and transformed using 10-base logarithm prior to analysis; CD4+ T-cell were compared to appropriate age and HIV-controls using Z-scores; Executive function and learning ability are compared to HIV– controls by Z-scores; HIV = human immunodeficiency virus; IQ and processing speed are compared with regular population; *p*-values were adjusted for age, sex, BMI;

Supplementary Table S2. Different cART regimens at first and second assessment in PHIV adolescents

cART Regimens	PHIV (n = 19)
Protease inhibitors (PIs)	
First assessment	7 (41%)
Second assessment	4 (24%)
Integrase strand transfer inhibitors (INSTIs)	
First assessment	1 (6%)
Second assessment	10 (58%)
Non-nucleoside reverse transcriptase inhibitors (NNRTIs)	
First assessment	9 (53%)
Second assessment	3 (18%)
Efavirenz	
First assessment	8 (47%)
Second assessment	3 (18%)
Ritonavir	
First assessment	6 (35%)
Second assessment	2 (11%)
Abacavir	
First assessment	10 (58%)
Second assessment	8 (47%)

Abbreviations: Values are reported in number and percentage (No %). PHIV (n = 19), with one missing value in first and second assessment; cART = Combination antiretroviral therapy; INSTIs = integrase strand transfer inhibitors; PIs = protease inhibitors; NNRTIs = non-nucleoside reverse transcriptase inhibitors;

Supplementary Table S3. Different cART regimens and association with pNfL and cognitive function variables over time

Association analysis of different cART regimens						
	**PIs exposure**	**INSTIs exposure**	**NNRTIs exposure**	**Efavirenz exposure**	**Ritonavir exposure**	**Abacavir exposure**
	Group x time	Group x time	Group x time	Group x time	Group x time	Group x time
**Executive function** #						
Coefficient (95% CI)	-0.3 (-1.1 – 0.5)	0.5 (-0.2 – 1.9)	0.6 (-0.2 – 1.3)	0.6 (-0.1 – 1.3)	-0.5 (-1.3 – 0.4)	0.3 (-0.7 – 1.3)
p-value	0.418	0.174	0.113	0.116	0.244	0.525
**Processing speed** #						
Coefficient (95% CI)	0.09 (-0.01 – 0.2)	-0.07 (-0.1 – 0.005)	0.01 (-0.06 – 0.09)	0.03 (-0.05 – 0.1)	0.03 (-0.06 – 0.12)	-0.04 (-0.1 – 0.03)
p-value	0.075	0.086	0.743	0.424	0.533	0.246
**Learning ability** #						
Coefficient (95% CI)	-0.1 (-0.9 – 0.7)	-0.3 (-0.7 – 0.5)	-0.4 (-1.0 – 0.3)	-0.2 (-0.9 – 0.5)	0.7 (-0.2 – 1.4)	-0.1 (-0.7 – 0.5)
p-value	0.789	0.489	0.222	0.547	0.093	0.706
**Visual motor function** #						
Coefficient (95% CI)	-0.13 (-0.08 – 0.3)	-0.15 (-0.3 – 0.04)	0.04 (-0.2 – 0.2)	0.09 (-0.1 – 0.3)	0.08 (-0.2 – 0.3)	-0.08 (-0.3 – 0.09)
p-value	0.256	0.135	0.647	0.392	0.513	0.391
**Working memory** #						
Coefficient (95% CI)	-0.001 (-0.3 – 0.3)	-0.09 (-0.4 –0.19)	0.09 (-0.2 – 0.4)	0.1 (-0.2 – 0.4)	-0.03 (-0.03 – 0.4)	0.05 (-0.2 – 0.3)
p-value	0.994	0.556	0.523	0.525	0.849	0.695
**Intelligence Quotient**						
Coefficient (95% CI)	-0.5 (-9.0 – 7.8)	-0.8 (-7.8 – 6.7)	1.1 (-6.0 – 8.3)	3.0 (-4.3 – 10.2)	-5.2 (-13.5 – 3.1)	2.8 (-2.6 – 8.5)
p-value	0.911	0.834	0.762	0.428	0.240	0.332
**pNfL (log pg/ml)** #						
Coefficient (95% CI)	0.17 (-0.3 – 0.7)	0.09 (-0.5 – 0.6)	-0.3 (-0.7 – 0.3)	-0.2 (-0.7 – 0.3)	0.4 (-0.2 – 0.9)	0.03 (-0.4 – 0.04)
p-value	0.520	0.755	0.347	0.427	0.254	0.910

Abbreviations: pNfL = plasma Neurofilament light; 95% CI = 95% Confidence Interval; Group x time = group by time interaction; INSTIs = integrase strand transfer inhibitors; PIs = protease inhibitors; NNRTIs = non-nucleoside reverse transcriptase inhibitors; Executive function and learning ability are compared to HIV- controls by Z-scores; Visual motor function, working memory, IQ and processing speed are compared with regular population; CD4+ T-cell were compared to appropriate age and HIV- controls using Z-scores. Linear mixed models' formulas were used for longitudinal association analysis. # These variables were transformed using natural logarithm prior to analysis.
